# Topical Iron Chelator Therapy: Current Status and Future Prospects

**DOI:** 10.7759/cureus.47720

**Published:** 2023-10-26

**Authors:** Tanya Ramadoss, Derek S Weimer, Harvey N Mayrovitz

**Affiliations:** 1 Medical School, Nova Southeastern University Dr. Kiran C. Patel College of Allopathic Medicine, Fort Lauderdale, USA; 2 Medical Education, Nova Southeastern University Dr. Kiran C. Patel College of Allopathic Medicine, Fort Lauderdale, USA

**Keywords:** ultra violet radiation, deferoxamine, skin damage, inflammation, wound healing, skin, iron, iron chelation, dermatology

## Abstract

Systemic iron chelation therapy has long been used for iron overload, providing a role in returning iron levels to proper homeostatic concentrations. Recently, topical iron chelation therapy has emerged as a potential strategy for treating skin damage. This narrative review explores the current status and future prospects of topical iron chelation therapy for treating ultraviolet (UV) and non-UV skin damage, as well as its potential application in wound healing. The review was conducted through a literature search across PubMed, Web of Science, and EMBASE databases, spanning publications from 1990 to 2023. The selection of articles was focused on primary research studies, either experimental or clinical, that explored the implications and formulations of topical iron chelators used alone or in conjunction with another therapeutic agent. The search strategy employed a combination of terms, including "topical iron chelation", “topical deferoxamine”, "UV”, “wound healing", "skin inflammation", “radiation-induced fibrosis”, and "skin cancer". Relevant studies, including methods, intervention strategies, measured outcomes, and findings, are summarized. The review also considered the potential challenges in translating research findings into clinical practice. Results indicate that topical iron chelators, such as deferoxamine, are effective in mitigating UV-induced skin damage, reducing tumorigenesis, and decreasing oxidative damage. In addition, the use of these agents in radiation-induced fibrosis has been shown to significantly increase skin elasticity and reduce dermal fibrosis. Several studies also highlight the use of topical iron chelators in difficult-to-treat chronic wounds, such as diabetic neuropathic ulcers and sickle cell ulcers. In conclusion, topical iron chelation therapy represents a novel and promising approach for skin protection and wound healing. Its potential makes it a promising area of future research.

## Introduction and background

Maintaining a proper balance of iron within the body is one of the key components of maintaining optimal health. Despite this, many people are either deficient or overloaded in this one simple element [1]. Optimum iron stores are approximately 38 and 50 mg iron/kg of body weight for adult women and men, respectively [1]. Primarily supplied by meat products and, to a lesser extent, plant products, iron is an important mediator in oxygen transport, muscular myoglobin functionality, and overall mitochondrial function within cells, with an array of clinical implications if iron levels are too low or too high [1]. One aspect relates to iron’s ability to create oxidative stress if iron levels are too high thereby causing excess generation of free radicals associated with the Fenton Reaction [2]. In this reaction, iron is used as a catalyst for creating hydroxyl free radicals from reactive oxygen species (ROS). This free radical formation, though not solely generated from iron, often leads to cellular destruction as seen in patients with iron overload as might occur secondary to iron supplementation or with pathological conditions such as hemochromatosis [2]. With overload, the iron is eventually stored within various tissues causing localized damage [3]. At the other extreme is iron deficiency which can also lead to an array of pathological conditions, including iron-deficiency anemia [4]. Causes of such deficiencies include decreased intestinal absorption, low iron diet, and proton pump inhibitor use [4-6]. Depletion of iron stores within the body can lead to symptoms of iron-deficiency anemia including dizziness, conjunctival pallor, and fatigue [1]. It is evident from these few examples that iron homeostasis is a critical process needed to limit oxidative stress and help minimize cellular destruction and tissue degradation.

The importance of maintaining iron homeostasis is reflected by the normal regulatory mechanisms for limiting free radical formation and controlling iron elimination. Some examples of components involved in this process include superoxide dismutase, catalase, glutathione, thioredoxin, and ferritin [7]. While antioxidants work to reduce ROS, iron chelators reduce iron availability [1]. The flavonoid antioxidant curcumin, for example, chelates iron, and regulates free radical formation by reducing lipopolysaccharide (LPS)-induced inflammatory reactions [1,8]. In addition, the increased inflammation caused by these reactions increases hepcidin expression, an iron-regulating hormone responsible for sequestering iron molecules within cells. When administering curcumin to mice, repression in both hepcidin and liver ferritin was seen [1,8]. This suggests curcumin has a role in iron chelation and suggests the possibility of a more general importance of iron chelation when considering inflammation.

Iron chelation therapy is far from novel. Chelating agents have been used clinically since the 1980s, primarily for the treatment of iron overload secondary to thalassemia intermedia [9]. Currently, there are three iron-chelating agents approved by the Federal Drug Administration (FDA): deferoxamine (DFOA)/desferrioxamine (DFO), deferiprone (DFP), and deferasirox (DFX) [10]. Though mechanistically different, the goal of iron chelation therapy is to reduce overall concentrations of iron within the body. This can be achieved by binding non-transferrin-bound (i.e., free) iron or by binding iron that is stored in ferritin, such as seen in DFO [11]. Binding iron in these forms later produces a molecule that is readily excreted via the kidneys [10,12]. Decreasing iron concentrations within the body reduces the risk of developing iron overload and subsequent free radical formation. On the contrary, adverse reactions with systemic iron chelators center around the concept of dangerously low levels of iron. Though different across formulations, serious, yet rare, adverse reactions include reversible neurological symptoms (e.g., visual and auditory disturbances) and bone abnormalities, seen primarily in patients on systemic use with high doses [10]. More common side effects include increased serum creatinine, gastrointestinal effects (e.g., nausea, vomiting, diarrhea, abdominal pain), allergies, and infusion site reactions (i.e., local inflammation), relating to the drug itself rather than the iron concentration with a resolution after discontinuation of the drug [10]. Despite a major role in decreasing iron-related destruction, FDA-approved iron-chelating agents are currently only utilized for systemic use (administered intravenous (IV), intramuscular, subcutaneous, and orally) [10,13]. Based on prior reports, the application of iron-chelating agents could be of use when considering skin inflammatory processes.

Even since the 1990s, iron has been studied in skin inflammatory reactions [14]. Skin is particularly susceptible to free radical formation due to exposure to various elements. For example, mechanical abrasion within the dermal layers generates a wound characterized by various immune mediators such as macrophages and polymorphonuclear leukocytes. Though providing endogenous immune protection, acute inflammation can sometimes lead to localized skin damage secondary to the release of ROS via respiratory burst reactions [1,14]. Additionally, skin is exposed to an array of environmental factors (e.g., ultraviolet (UV) radiation) that contribute to the formation of ROS with subsequent oxidative stress and inflammation [14,15]. This highlights the importance of considering iron-induced damages when studying underlying contributors involved in skin inflammatory processes.

## Review

The goal of this narrative review is to summarize and discuss the current understanding and potential use of topical iron chelation therapy to treat skin inflammation, damage, and wounds.

Search strategy

A literature search was conducted across PubMed, Web of Science, and EMBASE databases, spanning publications from 1990 to 2023. The search strategy employed a combination of terms, notably: "topical iron chelation," "transdermal deferoxamine," “topical lactoferrin," “topical deferoxamine,” “skin health," "UV,” “wound healing," "skin inflammation," “wound,” “ulcer,” “skin,” “radiation-induced fibrosis,” and "skin cancer." The following combination was searched: ((topical iron chelation) OR (transdermal deferoxamine) OR (topical lactoferrin) OR (topical deferoxamine)) AND ((skin health) OR (UV) OR (wound healing) OR (skin inflammation) OR (wound) OR (ulcer) OR (skin) OR (radiation-induced fibrosis) OR (skin cancer)). The initial search yielded 111 articles. These were further reviewed for specific relevancy and their potential inclusion in the current review. The selection of articles was focused on primary research studies, either experimental or clinical, that explored the implications and formulations of topical iron chelators used alone or in conjunction with another therapeutic agent.

Iron chelation for UV skin damage

Iron in the skin is a catalyst for UV-induced generation of ROS, which contributes to skin cancer, photosensitization, and photoaging, among many other negative consequences [16]. Considering iron's role in such skin manifestations, it calls to question whether iron-chelating agents should be integrated into skincare and maintenance. Preclinical models have demonstrated that topical iron chelators reduce UV-induced erythema, inflammatory cell production, and epidermal hyperplasia [17,18]. The action of topical iron chelators would appear beneficial for UV damage due to their effect on decreasing sunburn cells [17,18]. Sunburn cells are keratinocytes that undergo apoptosis due to irreversible and severe DNA damage following UV exposure [19]. By decreasing the concentrations of sunburn cells, the skin is protected from further UV-induced damage. In addition to providing UV protection, topical iron chelators limit skin tumorigenesis by reducing concentrations of ornithine decarboxylase, an enzyme involved in oncogenesis [17,18,20]. Furthermore, dermal inflammatory cells such as monocytes and epidermal keratinocyte overproduction are linked to the progression of skin photoaging and cancer development [21,22]. Considering the role of topical iron chelators in reducing inflammatory cells, prevention or delay of skin photoaging and cancer development is observed [16-18].

One of the iron chelator agents in current use is 2-furildioxime (FDO) which has shown utility in mitigating simulated solar UV effects [18]. In the study, mice, guinea pigs, and human skin biopsies were examined following simulated solar UV radiation to replicate radiation-induced erythema. UV exposure was simulated with a solar simulator equipped with a xenon arc lamp and dichroic filter, which produced a spectrum between 290 nm and 400 nm. The wavelength of UV radiation lies in the range of 100 nm to 400 nm [23]. In the experimental group, pretreatment of the skin with a 5% solution of FDO was conducted, followed by exposure to UV radiation and tissue sample retrieval. In mice, it was found that pretreatment with a single dose of 5% FDO administered two hours before UV exposure provided 90% protection against the induction of ornithine decarboxylase. Skin biopsies from 31 adult males indicated that three topical applications of 5% FDO administered 26, 18, and 2 hours before UV exposure, resulted in 0.15 sunburn cells/mm compared to 6.80 cells/mm as exhibited in the vehicle control group. The study also found that topical FDO prevented increases in dermal inflammatory cells measured 72 hours after UV exposure and prevented an increase in epidermal hyperplasia. Findings such as these suggest the possibility of wider use of iron-chelating agents for skin photoprotection [16,24].

In addition to FDO as a monotherapy, its use in combination with other photoprotective agents, such as sunscreens, has been explored [25]. Guinea pigs were utilized as test subjects to determine the sun-protecting capabilities of topical iron-chelating agents. It was found that using 5% FDO as a monotherapy provided a sun protection factor (SPF) of 3.5. However, when combined with sunscreen with a SPF of 4, the combination provided an SPF greater than 30. In addition to the SPF protection, the same study determined using topical iron chelators in protection against tumor growth. This time using a mouse model, the team generated cohorts involving pretreatments with FDO, sunscreen, or both [25]. Tumor growth was evaluated using the average time to develop at least one tumor during continued sunlamp exposure with a peak UV emission of 315 nm. Topical treatments were administered two hours before each irradiation, which occurred three times weekly until the development of at least one tumor. Tumors were classified as circular, red, raised, skin lesions larger than one mm in diameter. With FDO monotherapy, tumor onset was eight weeks and with sunscreen monotherapy, tumor onset was 12 weeks. When the FDO and sunscreen agents were used together, the tumor onset increased to 58.2 weeks, indicating a strong protective capacity when used in combination.

Kojic acid, a compound derived from *Aspergillus *and *Penicillium *fungi, is both an iron-chelating agent and an antioxidant used to mitigate UV-induced injury [26]. When compared to FDO and 1,10-phenanthroline, another iron-chelating agent, kojic acid was superior [17]. Utilizing a hairless mouse model, UV radiation was introduced for two hours per day, five times per week over 20 weeks. Dorsal skin samples were collected at the end of the irradiation period for evaluation of skin wrinkling, epidermal hyperplasia, adipose-to-fibrotic tissue conversion, and extracellular matrix components. Compared to vehicle controls, parameter negative changes were reported to be less in the presence of any of the chelating agents.

Iron chelation for non-UV skin damage

Radiation-related cancer treatments can increase inflammation, fibroblast activation, extracellular matrix deposition, and ROS generation [27-29]. Radiation-induced fibrosis is a progressive complication characterized by skin pigmentation, reduced elasticity, dermal thickening, cosmetic deformity, and pain [30-35]. Transdermal administration, compared to injection, of the iron chelator DFO in immunodeficient mice was found to decrease radiation-induced oxidative damage, dermal thickness, and collagen content, while increasing skin elasticity and vascularity, ultimately reducing dermal fibrosis [36]. In this study, immunodeficient mice were exposed to radiation for six sessions every other day for two weeks in the form of an X-ray. Mice received DFO through a transdermal patch or injection every day either prophylactically, during the acute injury phase (two-week irradiation regimen with four weeks of recovery), during the post-recovery chronic injury phase, or continuously through the entire experiment. Oxidative stress was determined by measurements of 8-isoprostane levels, a marker for ROS known to rise in response to radiation injury, and the ratio of oxidized to reduced glutathione. It was found that both topical and injected DFO agents were effective in mitigating adverse effects secondary to radiation. Though beneficial, the topical agent used provided an even greater reduction in the ratio of the glutathione oxidized to reduced forms thus exemplifying a potential benefit in radiation-induced damage. With DFO injections, it was observed that administration in the same area caused injury that is considered counterproductive to wound healing and increased the risk for bacterial infection, which may cause attendant inflammation and promote subsequent fibrosis. In addition, topical DFO was found to ameliorate dermal fibrosis and augment dermal blood supply in the prophylaxis, acute injury phase, chronic injury phase, and continuous groups. Notably, continuous therapy yielded the greatest results.

In a similar study, prophylactic topical DFO was administered two weeks prior to irradiation therapy in immunodeficient mice [37]. Immediately following irradiation, ROS and apoptotic markers, such as the protein Bax, Cleaved Caspase-2, and Dk1+ fibroblasts, were significantly decreased in comparison to a control group with no pretreatment but similarly irradiated [38-40]. In this study, skin ROS was assessed based on the levels of dihydroethidium, since it is a molecular probe for free radicals [41]. The finding of decreased ROS markers attributable to the DFO pretreatment further supports its consideration of iron-chelating agents in subjects that will be exposed to radiation therapy.

Iron chelation for protection against environmental pollution effects

Particulate matter resulting from pollution contributes to skin damage, especially debris less than 10 µm in diameter including dust, industrial emissions, and automobile emissions [42]. For example, diesel engine exhaust contains polycyclic aromatic hydrocarbons that can be readily absorbed through the skin and damage cellular mitochondria [43]. When exposed to diesel exhaust, the skin’s stratum corneum lipids are oxidized, forming products like 4-hydroxynonenal (4HNE), leading to subsequent skin damage. Utilizing human skin biopsies, one study determined the efficacy of DFO in combination with the topical antioxidant CE Ferulic, a combination of vitamin C, vitamin E, and ferulic acid. After application to human skin biopsies at 24 hours and repeated 30 minutes prior to exposure to diesel exhaust, 4HNE concentrations were determined [43]. Diesel engine exhaust was generated by a diesel engine which was on for 10 seconds in an exposure chamber before skin explants were left in the sealed chamber for 30 minutes. Treatment and exposure cycles were performed daily over four days. Compared to untreated tissue, the combined therapy significantly suppressed 4HNE levels (p < 0.05), measured using fluorescent intensity upon staining.

Iron as a factor in delayed wound healing

Chronic wounds can be defined as, “wounds that have not proceeded through an orderly and timely reparation to produce anatomic and functional integrity after three months” [44]. Largely characterized by diminished oxygen tension secondary to compromised local vasculature, iron has been suggested to play a contributory role in delaying wound recovery [45]. For example, the regulatory protein complex, hypoxia-inducible factor 1 (HIF-1), is responsible for activating genes involved in tissue repair, cell growth, cell proliferation, and angiogenic factors such as vascular endothelial growth factor (VEGF), all critical for proper wound healing. When sufficient oxygen is available, HIF-1 is downregulated due to forming a complex with propyl 4-hydroxylase, an enzyme that requires iron as a cofactor. On the contrary, when hypoxia ensues, iron levels become reduced allowing HIF-1 to stabilize and forward the wound healing process [45].

In addition to iron’s role in oxygen homeostasis, studies have shown iron’s role in inflammation when considering chronic wound pathogenesis. In chronic venous ulcers, for example, significantly increased iron deposits were found, highlighting a possible pro-inflammatory role for iron, particularly in white blood cell recruitment [46-51]. Another study analyzing 1064 obese individuals with a history of bariatric surgery found that serum iron and transferrin saturation were increased in patients with major postoperative complications, particularly wound c­omplications [52]. Treatment of chronic wounds is often difficult due to high levels of pain, and resistance to a variety of conventional therapies, and are many times recurrent in nature [53]. A diabetic neuropathic pressure ulcer, for example, is a chronic complication of diabetes mellitus seen clinically yet often poorly controlled [54].

Iron chelation for wound healing

Iron chelation as a form of either prophylaxis or treatment has been investigated in several wound types in experimental animals.

Diabetic Neuropathic Ulcers

The topical application of DFO, a HIF-1 inducer and stabilizer, has been studied as a therapeutic approach to wound healing. In one study, a transdermal delivery system, utilized due to DFO’s hydrophilic nature and shorter half-life, was created to determine its effect on wound healing of pressure-induced diabetic ulcers [55]. In mice, a pressure-induced ulcer was made by placing a ceramic magnet on both sides of dorsal skin for seven days with six hours on and six hours off. Transdermal DFO was applied 24 hours after the last cycle and the patch was changed every 48 hours until complete ulcer healing. The DFO-treated mice had an average wound closure time of 27 days compared to 39 days in the untreated control group. The treated group showed increased expression of VEGF and enhanced neovascularization. In addition to utilization as a treatment option, the same study demonstrated topical DFO was effective prophylactically in preventing diabetic ulcers and skin necrosis. This was demonstrated by pretreating the dorsal skin for 48 hours with the transdermal DFO, followed by removal of the transdermal DFO and measured ulcer induction. In mice pretreated with transdermal DFO, ulcer formation and skin necrosis were prevented across all three days observed. The control group with no pretreatment formed ulcerations on day 0 of ulcer induction, measured by wound area and histological sections. Other studies have observed similar results when evaluating the efficacy of DFO in preventing or treating pressure-induced diabetic ulcers in mice [56,57].

Another potent iron chelator that has been studied for its use in treating diabetic pressure ulcers is a recombinant form of human lactoferrin named talactoferrin alpha [58-60]. In 55 diabetic patients with foot ulcerations, patients treated with either 2.5% or 8.5% talactoferrin gel twice daily for 12 weeks had twice the incidence of significant ulcer size reduction, defined as ≥75% reduction in ulcer size, compared to the control group treated with a placebo gel [58]. Notably, no talactoferrin-related adverse events for both doses were reported.

Sickle Cell Ulcers

The positive findings attributed to iron chelation in diabetic ulcers suggest the possibility of its use in other types of skin wounds. Sickle cell ulcers (SCUs) arise as a complication affecting patients with sickle cell disease [53]. Similar to pressure-induced diabetic ulcers, SCUs can also cause chronic pain and are more prevalent in the foot, leading to long-term deformities [61]. To date, there is no uniformly effective therapy for SCUs highlighting the need for innovative therapeutic options [53]. One such study used a mouse model of SCU in which the effects on healing of transdermal DFO, injected DFO, and no treatment was compared [61]. Outcomes were assessed by the time required for complete ulcer closure which was reported as 13, 14.5, and 17.1 days respectively. The differences in wound area among the groups were statistically significant from day 6 until closure. Wound remodeling was evaluated through histological examination of the healed wounds and showed that the DFO treated had increased collagen deposition in the dermis. One limitation noted in topical DFO use is limited penetration into the stratum corneum. However, in skin ulcers in which the DFO is applied directly to the wound bed, this is not a limitation [62].

Irradiated Skin

In addition to ulcerations, the use of topical iron chelation therapy can be implemented for wound healing in irradiated skin. In one study, excisional wounds were created on irradiated dorsal skin surfaces of mice and were treated with either a DFO-containing patch or a patch without DFO [63]. The average open wound area measured 14 days following wound induction was 10.34% for the DFO-treated group and 18.71% for the untreated group. This finding suggests that topical DFO may have a potential role in improving the healing rate in irradiated skin, providing symptomatic resolution within a shorter duration. Furthermore, on day 21, the untreated group showed 30% failure in wound closure whereas the DFO-treated group demonstrated only a 10% failure. The study’s authors concluded that the increased efficacy and speed of wound healing of topical DFO may be partly related to DFO’s ability to potentiate the activity of nitric oxide in irradiated wounds. This conclusion follows from the fact that nitric oxide induces collagen deposition [64].

Discussion

Iron-chelating agents are far from novel, however, their use in topical formulations is a rather new concept [26]. Though research is limited, preliminary studies have described efficacy when testing the use of topical iron chelation therapy in UV-induced, non-UV-induced skin damage, and wound care.

When considering UV-induced skin damage, photoprotection centers around primary and secondary factors [65]. Primary factors include physical barriers that reflect and scatter light and chemical barriers, such as organic sunscreens, that absorb high-energy UV rays. Secondary factors include antioxidants, osmolytes, and DNA repair enzymes, which function by disrupting the photochemical cascade in order to limit skin damage caused by sunlight. In this context, topical iron chelators similarly disrupt the photochemical cascade, also protecting UV-induced skin damage. They achieve this by reducing the generation of harmful ROS, erythema, sunburn cell formation, and dermal inflammatory cell production, similar to existing secondary factors exemplified in sunscreen [18]. As mentioned previously, the use of topical iron chelators in conjunction with traditional sunscreen has shown great benefit. By creating a combined formulation, a synergistic effect increased the SPF 4 sunscreen to an SPF of greater than 30 [25]. The mentioned study also displayed many promising results including delayed tumor and wrinkle formation.

Though promising preliminary results, topical iron-chelating agents need to be explored longitudinally. One consideration for longitudinal studies should be aimed at potential side effects. To note, adverse effects pertaining to ocular, auditory, and neurological disturbances, have been reported with the use of subcutaneous DFO administration, but none have been explored in topical use of DFO or other iron-chelating agents [58,62]. Iron deficiency anemia may present with pallor, pruritis, predisposition to skin infections, and other cutaneous manifestations [26]. It has not been explored if cutaneous deficiency of iron may have similar side effects. A deeper investigation into this area is warranted prior to progression to human clinical trials. Another consideration for longitudinal studies is combination therapy. Several studies have used topical iron chelators in conjunction with antioxidative agents or used compounds that have both iron chelating and radical scavenging properties [17,43]. Though demonstrating the short-term benefits of using topical iron chelators in combination with other protective compounds to reduce wrinkling and cutaneous inflammation, little is known about their long-term benefits.

As a standalone therapy, topical iron chelators have been explored for their use in radiation-induced fibrosis and wound healing. Radiation-induced fibrosis is a serious complication of radiation-related cancer treatments and current therapy relies largely on symptomatic control, with no effective method that offers complete remission currently [66]. Studies have illustrated the use of DFO in decreasing oxidative damage, increasing skin elasticity and vascularity, and ultimately reducing dermal fibrosis induced by radiation [36]. In this study, it is important to note that the transdermal DFO group was more efficacious than DFO injections in reducing oxidative stress, as demonstrated by the oxidized to reduced glutathione ratio. Moreover, the prophylactic use of topical DFO was found to reduce radiation-induced fibrosis, highlighting a potential therapy for this disease [37]. Given the limited treatment options available and the low efficacy of traditional therapy, topical iron chelation should be considered as a potential avenue for achieving benefit in these patients.

Another potential use of topical iron chelators as a standalone therapy is wound healing, specifically in ulcers that are notoriously difficult to treat such as those relating to sickle cell disease and diabetes mellitus. Topical application of DFO showed significantly improved wound closure time, enhanced neovascularization, and increased collagen deposition in the dermis [61]. Though efficacy may be elevated due to the lack of a stratum corneum in open ulcers, significant improvement was observed making topical iron chelation a consideration in difficult-to-treat ulcerations in patients refractory to other treatments.

Lastly, a majority of the existing topical iron chelation therapy studies were not conducted with human subjects. Instead, these studies were conducted on animal models, primarily mice. Compared to murine models, the human skin contains more epidermal layers, which may present difficulty in transitioning from animal to human subjects [67]. It is known that topical delivery of iron chelators such as FDO is complicated by its relatively high atomic mass and hydrophilicity, contrasting with the lipophilic stratum corneum layer of the skin. This reiterates the difficulty of creating formulations as higher lipophilicity is typically associated with greater absorption. To combat this potential difficulty, the development of a transdermal DFO delivery system has been shown to be effective. DFO encapsulated in reverse micelles, permits the delivery of DFO through the stratum corneum, allowing for a therapeutic effect in the upper skin layers. In addition to superficial layers, this delivery system has also been shown to penetrate the deeper dermal layers as well within 24 hours of use [55]. With the invention of promising delivery methods for topical iron chelators and exhibited efficacy in animal models, further exploration into the potential uses and adverse effects of topical iron chelators is certainly warranted.

## Conclusions

Topical iron-chelating agents have emerged as a promising therapy for skin protection and wound healing. Despite its somewhat novel application, preliminary studies have demonstrated the efficacy of these agents in reducing UV-induced skin damage and radiation-induced fibrosis, as well as improving wound healing. The potential for topical iron chelators to be used as a standalone therapy in radiation-induced fibrosis and wound healing, as well as in combination with other protective compounds, highlights the vast potential of this therapy. The development of innovative delivery methods for topical iron chelators has also added to the complexity surrounding this area of research. Although further research is necessary to fully understand the potential adverse effects and efficacy of topical iron chelators in human subjects, the promising results from preliminary studies make this an exciting area for future investigation.
